# Investigation of the effectiveness Of ‘Nordic Walking’ training on geriatric individuals: a randomized comparative study

**DOI:** 10.1007/s41999-026-01507-w

**Published:** 2026-05-19

**Authors:** Nursel Öziri, Emre Baskan, Berna Cagla Balkisli

**Affiliations:** 1 Istanbul Rumeli University, Physiotherapy and Rehabilitation, Istanbul, Turkey; 2 Pamukkale University, Physiotherapy and Rehabilitation, Denizli, Turkey; 3 Istanbul Okan University, Physiotherapy and Rehabilitation, Istanbul, Turkey

**Keywords:** Geriatrics, Nordic walking, Physical activity, Quality of life

## Abstract

**Purpose:**

This study aimed to investigate the effects of Nordic Walking training on muscle strength and endurance, balance, falls and fear of movement, aerobic capacity and quality of life in older individuals.

**Methods:**

A total of 32 individuals aged 65–80 years were randomly assigned to either the Nordic Walking (NW) group or the Traditional Walking (GY) group. Outcomes included the 30-s sit-to-stand test (STS), 30-s biceps curl test (BCT), timed up and go test (TUG), 6-min walk test (6-MWT), Tinetti falls efficacy scale (TFES), Tampa kinesiophobia scale (TKS), and World Health Organization Quality of Life–Older Adults Module (WHOQOL-OLD). All outcomes were assessed at baseline and after a 12-week supervised training program (3 sessions/week).

**Results:**

Of the 32 participants enrolled, 28 completed the study (NW—*n* = 14; TW—*n* = 14). Both groups improved across outcomes, with greater improvements in the NW group for STS (MD: 1.36; 95%CI 0.48–2.24), BCT (left—MD: 2.33; 95%CI 1.37–3.28; right—MD: 2.13; 95%CI 1.17–3.09), 6-MWT (MD: 51.13 m; 95%CI 23.78–78.47), TFES (MD: − 3.49; 95%CI  − 5.35 to  − 1.65), TKS (MD:  − 8.08; 95%CI − 12.32 to − 3.84), and WHOQOL-OLD (MD: 3.46; 95%CI 0.84–6.09). No significant between-group difference was observed for TUG.

**Conclusion:**

NW training was superior to traditional walking in improving parameters such as, upper and lower extremity muscle strength and performance, aerobic capacity, fall efficacy, fear of moving, and quality of life in older individuals. NW training is an effective method for geriatric rehabilitation programs.

**Clinical trial registration:**

NCT07158931.

## Key summary points

**Aim:**

To determine whether Nordic Walking training provides greater improvements than traditional walking in muscle strength, balance, falls and fear of movement, aerobic capacity, and quality of life among geriatric individuals.

**Findings:**

Nordic walking led to significantly greater improvements in lower and upper extremity strength, aerobic capacity, fall efficacy, kinesiophobia, and quality of life compared to traditional walking. Only the Nordic Walking group showed meaningful gains in upper-extremity endurance.

**Message:**

Nordic walking is a superior and effective rehabilitation option for enhancing physical and psychosocial outcomes in older individuals.

## Introduction

Aging is associated with progressive declines in aerobic capacity, muscle strength, and mobility, leading to functional impairments and increased risk of chronic disease in older adults [1, 2]. As physical function deteriorates, gait quality and safe walking capacity are compromised, resulting in reduced independence and quality of life. Regular physical activity plays a key role in promoting healthy aging by preserving functional abilities, reducing frailty and fall risk, and lowering morbidity [3, 4].

Walking is one of the most widely recognized forms of physical activity worldwide, accessible to people of all ages, including older adults [5]. In recent years, Nordic walking, defined as walking with the dynamic use of specially designed poles, has gained popularity as a form of exercise. Studies in the literature have investigated Nordic walking (NW) training in older adults, evaluating its effects on physiological and neuromuscular parameters. During Nordic walking, the trunk and upper extremities are actively engaged while the natural walking pattern is maintained. Since it involves greater muscle mass, NW induces a higher cardiovascular load compared to regular walking [6, 7]. In addition, the use of poles enhances stability, increases step length, and reduces the duration between foot contact and heel-off [8].

Despite these benefits, there is still a lack of studies directly comparing the effects of regular walking and Nordic walking on functional parameters, fall risk, and fear of moving in older adults. Therefore, the present study aimed to examine the effects of NW training on lower and upper extremity muscle strength and endurance, balance, falls and fear of movement, aerobic capacity, and quality of life in older individuals, and to compare these outcomes with those obtained through traditional walking.

## Methods

### Study design

This prospective, randomized controlled trial with parallel groups was conducted between October 2022 and March 2024 at the Physical Therapy and Rehabilitation Departments of Özel Cadde Medical Center and Pamukkale University Hospital. The study was conducted in accordance with the principles of the Declaration of Helsinki. Ethical approval was obtained from the Non-Interventional Clinical Research Ethics Committee of Pamukkale University (Decision No: 11, dated 26.07.2022), and the study was registered at ClinicalTrials.gov (Identifier: NCT- 07158931).

### Sample size

The sample size was determined by an a priori power analysis conducted using G*Power version 3.1. The statistical test selected was t-tests and means: difference between two independent groups (two groups), appropriate for comparing the post-intervention outcomes between the Nordic Walking and control groups. Based on systematic reviews and meta-analyses evaluating aerobic capacity including the 6-min walk test after Nordic Walking interventions compared to sedentary control groups, a large effect size (Cohen’s *d* = 0.92) was used. With an alpha level of 0.05 and statistical power set at 0.80, the analysis indicated that a minimum total of 32 participants (16 per group) was required to detect statistically significant between-group differences.

### Participant recruitment and randomization

A total of 32 community-dwelling older adults were included in the study. The participants were recruited from individuals who applied to the outpatient Physical Therapy and Rehabilitation Departments of Özel Cadde Medical Center and Pamukkale University Hospital. The inclusion criteria were: being between 65 and 80 years of age, having a score of 24 or above on the Standardized Mini-Mental State Examination (SMMSE), and voluntarily agreeing to participate in the study. Participants were eligible if they had stable symptoms and medication use and no active diseases affecting daily living activities.

Exclusion criteria included conditions that could compromise safe participation in walking exercise, such as a history of cerebrovascular accident with residual functional impairment, severe visual impairment affecting balance, active infection, malignancy under active treatment, multiple organ failure, or terminal illness. The participants were also excluded if they had a history of lower or upper extremity fracture within the last 3 months, or any musculoskeletal condition that would prevent safe engagement in exercise. In addition, individuals with neurological disorders affecting mobility, balance, or cognitive function, including Alzheimer’s disease, Parkinson’s disease, dementia, or benign paroxysmal positional vertigo (BPPV), were excluded. Participants who had engaged in regular exercise training (≥ 150 min per week) within the past 6 months were also excluded to ensure a relatively sedentary baseline population.

Recruitment was based on voluntary participation and convenience sampling from outpatient clinical and community settings. The participants were screened for eligibility according to the inclusion and exclusion criteria through face-to-face interviews conducted by a physiotherapist. During these interviews, detailed information about the study was provided both verbally and in writing, and each eligible individual received a written information sheet. Those who voluntarily agreed to participate signed the informed consent form and were scheduled for a second face-to-face appointment for baseline assessments. The participants were clearly informed that they could withdraw from the study at any time without any obligation. Following baseline assessments, participants were randomly assigned to either the Nordic Walking Group or the Traditional Walking Group in a 1:1 ratio using a computer-generated simple randomization sequence. The physiotherapist conducted participant enrollment, assessment, and intervention procedures, and group allocation was performed after completion of baseline assessments. Due to the nature of the study, participants, intervention provider, and outcome assessor were not blinded. However, the data analyst was blinded to group allocation during statistical analysis.

### Assessments

Assessments were conducted at baseline and after the 12-week intervention. Following the recording of demographic data, lower extremity muscle strength and endurance were evaluated using the sit-to-stand test, upper extremity muscle strength and endurance were assessed with the biceps curl test, aerobic capacity was measured using the 6-min walk test, and balance was evaluated with the Timed Up and Go Test. Fall efficacy was assessed with the Tinetti falls efficacy scale, fear of moving was measured using the Tampa Scale for Kinesiophobia, and quality of life was assessed using the World Health Organization Quality of Life Instrument–Older Adults Module.

### Primary outcome measure

6-Minute Walk Test (MWT): It is a widely used, simple, and validated clinical assessment to measure aerobic capacity and functional exercise tolerance in older adults [9]. Participants walked along a flat 30-m corridor and the total distance covered in meters was recorded.

### Secondary outcome measures

Sit-to-stand test (STS): This is a widely used clinical assessment tool in older adults for evaluating lower limb function, muscle strength, balance, and overall physical performance in older adults. The participants were instructed to stand up and sit down repeatedly from a standard chair for 30 s, and the total number of completed repetitions was recorded [10].

Timed Up and Go Test (TUG): This is a clinical and research tool to assess mobility, balance, walking ability, and fall risk in older adults [11]. Participants were instructed to stand up from a chair, walk 3 m, return, and sit down. The average time of two trials was recorded in seconds.

Biceps curl test (BCT): This is a widely used assessment in geriatrics to evaluate upper body strength and endurance, particularly of the arm muscles [12]. Participants performed repeated elbow flexion using standardized dumbbell weights (2 kg for women and 3 kg for men), and the total number of repetitions completed within 30 s was recorded.

Tinetti Falls Efficacy Scale (TFES): Fear of falling was assessed using the Tinetti Falls Efficacy Scale, a validated self-report measure of confidence in performing daily activities without falling. Higher scores indicate greater fear of falling [13].

Tampa Kinesiophobia Scale (TKS): It is a 17-item self-report questionnaire rated on a 4-point Likert scale, designed to assess fear of movement or re-injury. Higher scores indicate greater levels of fear of moving [14].

World Health Organization Quality of Life Instrument–Older Adults Module (WHOQL- OLD): It is a 24-item questionnaire assessed on a five-point Likert scale, covering six domains: sensory abilities, autonomy, past-present-future activities, social participation, death and dying, and intimacy. Each domain is scored from 4 to 20, and higher total scores indicate better quality of life [15].

### Interventions

Nordic and traditional walking interventions were individually supervised (1:1) and administered by a physiotherapist with 30 years of professional experience in geriatric rehabilitation. Physiotherapist walked alongside participants, providing continuous encouragement throughout the sessions. Training was conducted three times per week for 12 weeks, either indoors or outdoors depending on weather conditions.

### Nordic walking group

Participants completed a 1-week familiarization phase (three sessions per week) prior to the intervention to ensure safe and effective use of poles. Walking duration during this phase ranged from 15 to 20 min. During the intervention, exercise intensity was maintained at 40–60% of heart rate reserve using a pulse oximeter. Pole height was individually adjusted to achieve approximately 90° elbow flexion.

### Traditional walking group

Participants performed walking on flat surfaces (e.g., concrete or compacted soil) under the same supervision. Exercise intensity was maintained below 60% of maximal heart rate and adjusted to allow comfortable conversation during walking.

Each session comprised three phases: warm-up, walking, and cool-down. The standardized warm-up routine consisted of 1 min of slow-paced walking; 10 repetitions of heel raises (with or without support), seated ankle dorsiflexion, mini squats, and scapular adduction; and stretching exercises for the shoulders and wrists. Following each walking session, the cool-down protocol included 2 min of slow-paced walking and static stretching (20 s each) for the quadriceps, hamstrings, lumbar extensors, gastrocnemius, shoulder girdle, and wrist flexor and extensor muscles.

Exercise progression in both groups was achieved by increasing walking duration: 15–20 min (weeks 1–2), 30 min (weeks 3–4), and 50 min (weeks 5–12). Adherence was supported by rescheduling missed sessions within the same week, ensuring that all participants completed three sessions per week, for a total of 36 sessions.

### Statistical analysis

The data were analyzed using IBM SPSS Statistics Standard Concurrent User V26 software (IBM Corp., Armonk, New York, USA). Descriptive statistics were reported as means ± standard deviations for continuous variables frequencies (%) for categorical variables. Normality was assessed using the Shapiro–Wilk test. The baseline characteristics were summarized descriptively without formal statistical comparison. The analysis of intervention effects was conducted using analysis of covariance (ANCOVA) to compare post-intervention outcomes between groups, adjusting for baseline values, age, and sex. Adjusted mean differences with 95% confidence intervals (CI) and effect sizes (partial eta-squared, ηp^2^) were reported. Between-group comparisons of change (Δ) scores (after intervention – before intervention) were performed using the Mann–Whitney U test for descriptive purposes. A *p* value of < 0.05 was considered statistically significant.

## Results

**Fig. 1:**
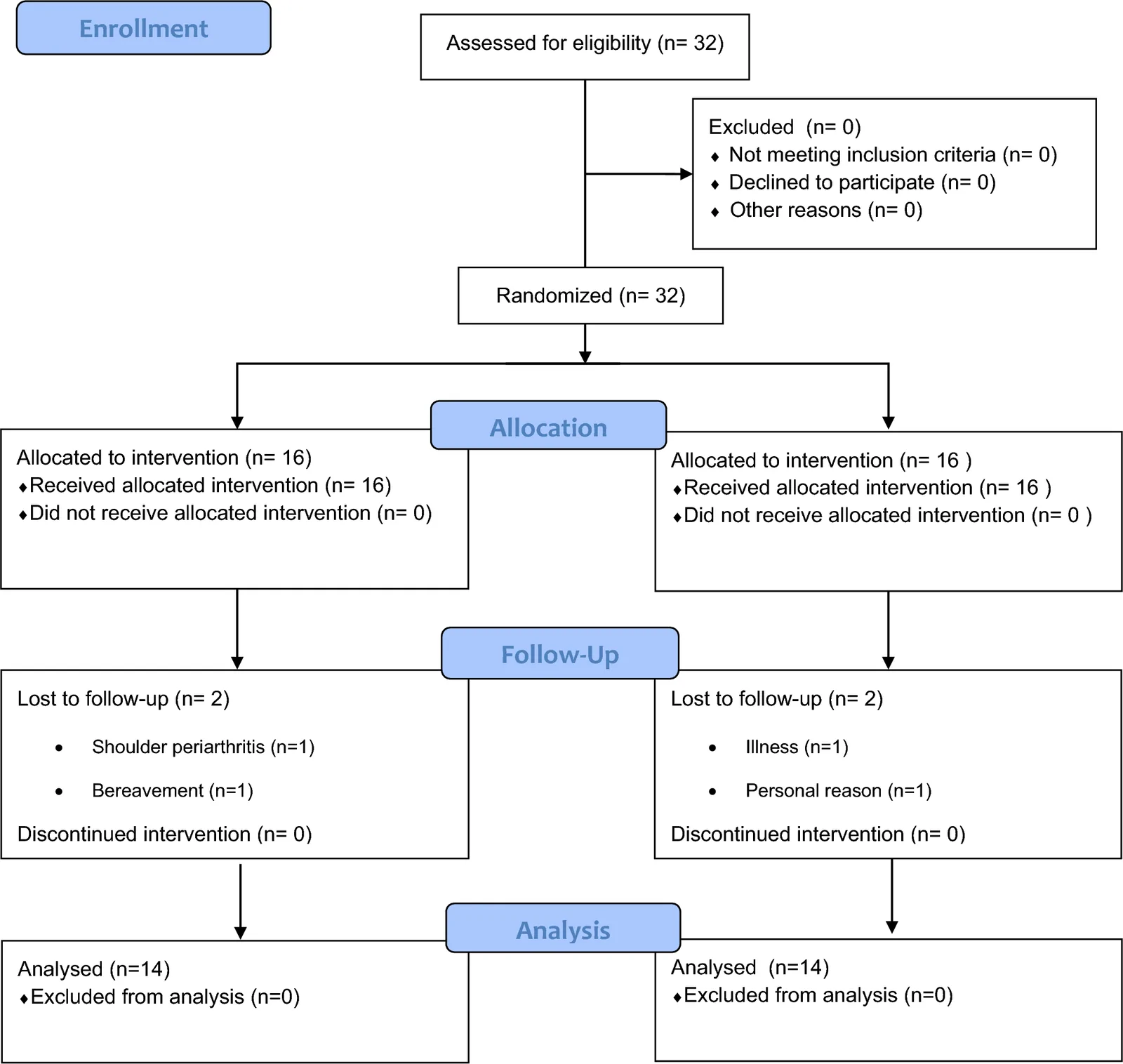
Flow diagram of study

A total of 32 participants who met the inclusion and exclusion criteria were enrolled and randomized in the study. Of these, 28 participants completed the intervention (NW: *n* = 14; TW: *n* = 14), corresponding to a retention rate of 87.5%. Four participants were lost during follow-up due to illness (*n* = 1), bereavement (*n* = 1), personal reasons related to session frequency (*n* = 1), and a newly diagnosed shoulder periarthritis (*n* = 1). No missing outcome data were observed among the participants who completed the study. The flow diagram of the study is shown in Fig. 1.

The baseline characteristics are presented in Table 1. The majority of participants were female (NW: 71.4%; TW: 64.3%), with a mean age of 70.6 years in the NW group and 72.5 years in the TW group. Anthropometric characteristics were broadly similar between groups. However, some baseline differences were observed. The NW group demonstrated higher baseline functional performance, particularly in the 6-MWT (392 m vs. 328 m), and better TUG scores (8.13 vs. 11.48 s) compared to the TW group.

**Table 1:** Descriptive characteristics of the participants in both groups

	Group	
	Nordic walking	Traditional walking
	*n* = 14	*n* = 14
Gender, *n* (%)		
Female	10 (71.4)	9 (64.3)
Male	4 (28.6)	5 (35.7)
Age, (years)		
* X* ± *SD*	70.64 ± 5.39	72.50 ± 5.96
* M* (*min*–*max*)	68 (65; 79)	75 (65; 80)
Height, (cm)		
* X* ± *SD*	160.50 ± 7.31	161.07 ± 10.59
* M* (*min*–*max*)	160.5 (150; 172)	160 (147; 182)
Body weight, (kg)		
* X* ± *SD*	70.50 ± 12.86	73.86 ± 14.70
* M* (*min*–*max*)	68.5 (54; 104)	73.5 (50; 104)
Body mass index, (kg/m^2^)		
* X* ± *SD*	27.52 ± 5.64	28.76 ± 6.62
* M* (*min*–*max*)	26.7 (20.1; 41.7)	27.8 (19; 42.7)
Marital status, *n* (%)		
* Married*	9 (64.3)	9 (64.3)
* Divorced/Widowed*	5 (35.7)	5 (35.7)
Educational status, *n* (%)		
* High school and below*	6 (42.9)	5 (35.7)
* University*	8 (57.1)	9 (64.3)
Chronic disease, *n* (%)		
* None*	7 (50)	6 (42.9)
* Present*	7 (50)	8 (57.1)
Standard Mini Mental Test		
* X* ± *SD*	28.93 ± 1.33	28.71 ± 1.98
* M (min–max)*	30 (27; 30)	30 (25; 30)

Statistically significant values (*p* < 0.05) are indicated in bold

Descriptive comparisons of pre- and post-intervention values and corresponding change (Δ = After intervention − Before intervention) scores are presented in Table 2. Both groups showed improvements across several outcomes. The NW group demonstrated larger changes than the TW group in STS, BCT (right and left), 6-MWT, TFES, TKS and WHOQOL-OLD. In contrast, changes in the TUG test were comparable between groups.

**Table 2:** Descriptive comparisons of pre- and post-intervention values and change (Δ) scores

	Group		
	Nordic Walking	Traditional Walking	
	*n* = 14	*n* = 14	
Sit to Stand Test (rep)			
Before	12.9 ± 2.8	11.8 ± 3.0	
After	15.6 ± 2.8	13.3 ± 2.9	
Delta ^&^ (After-Before)	2.8 ± 1.3	1.5 ± 0.8	***p* = 0.003 *r* = 0.576**
Biceps Curl Test-Right (rep)			
Before	16.5 ± 3.2	16.4 ± 4.5	
After	19.1 ± 3.5	16.7 ± 4.2	
Delta ^&^ (After-Before)	2.6 ± 1.5	0.4 ± 0.6	***p* < 0.001 *r* = 0.765**
Biceps Curl Test-Left (rep)			
Before	16.7 ± 3.0	16.5 ± 4.7	
After	19.4 ± 3.0	16.9 ± 4.4	
Delta ^&^ (After-Before)	2.6 ± 1.5	0.4 ± 0.6	***p* < 0.001 *r* = 0.785**
6-Minute Walk Test (m)			
Before	392.1 ± 61.1	327.6 ± 89.6	
After	499.6 ± 77.6	383.6 ± 101.8	
Delta ^&^ (After-Before)	107.6 ± 38.7	55.9 ± 28.5	***p* < 0.001 *r* = 0.681**
Timed Up and Go Test (s)			
Before	8.1 ± 2.9	11.5 ± 6.1	
After	6.8 ± 2.1	8.9 ± 3.3	
Delta ^&^ (After-Before)	− 1.3 ± 0.9	− 2.54 ± 3.3	*p* = 0.054 *r* = 0.379
Tinetti Falls Efficacy Scale			
Before	19.6 ± 16.1	17.7 ± 7.8	
After	12.9 ± 4.9	16.5 ± 6.6	
Delta ^&^ (After-Before)	− 6.7 ± 11.7	− 1.2 ± 2.0	***p* = 0.031 *r* = 0.423**
Tampa Kinesiophobia Scale			
Before	41.4 ± 6.1	42.9 ± 9.9	
After	29.6 ± 8.5	39.4 ± 8.1	
Delta ^&^ (After-Before)	− 11.8 ± 6.2	− 3.5 ± 5.3	***p* = 0.002 *r* = 0.599**
World Health Organization Quality of Life Instrument–Older Adults Module			
Before	74.7 ± 10.7	70.3 ± 8.7	
After	79.2 ± 8.6	71.8 ± 9.1	
Delta ^&^ (After-Before)	4.5 ± 4.4	1.5 ± 2.1	***p* = 0.027 *r* = 0.433**

Effect size (r), Delta: After intervention − Before intervention (&). Descriptive statistics are reported as mean (X̄) and standard deviation (SD). Bolded sections indicate statistically significant differences (*p* < 0.05)

The adjusted analysis of intervention effects, controlling for baseline scores, age, and sex, is presented in Table 3. The NW group demonstrated greater improvements than the TW group across several outcomes. Specifically, the adjusted mean difference (MD) for the STS was 1.36 (95% CI: 0.48 to 2.24), for the BCT was 2.33 (95% CI: 1.37 to 3.28) on the left and 2.13 (95% CI: 1.17 to 3.09) on the right, and for the 6-MWT was 51.13 m (95% CI: 23.78 to 78.47).

**Table 3:** ANCOVA results for between-group comparisons of outcome measures adjusted for baseline scores, age, and sex

Outcome measure	Adjusted mean difference (B)	95% confidence interval	*p* value	Partial Eta Squared (ηp^2^)
Sit to Stand Test (rep)	1.36	[0.48, 2.24]	**0.004**	0.30
Biceps Curl (Left, rep)	2.33	[1.37, 3.28]	** < 0.001**	0.52
Biceps Curl (Right, rep)	2.13	[1.17, 3.09]	** < 0.001**	0.48
6-Minute Walk Test (m)	51.13	[23.78, 78.47]	** < 0.001**	0.41
Timed Up and Go (sec)	− 0.40	[− 1.15, 0.36]	0.288	0.05
Tinetti Falls Efficacy Scale	− 3.49	[− 5.35, − 1.65]	** < 0.001**	0.40
Tampa Kinesiophobia Scale	− 8.08	[− 12.32, − 3.84]	** < 0.001**	0.40
WHOQOL-OLD	3.46	[0.84, 6.09]	**0.012**	0.24

Statistically significant values (*p* < 0.05) are indicated in bold

For the TFES, the adjusted mean difference was − 3.49 (95% CI: − 5.35 to − 1.65), and for the TKS, it was − 8.08 (95% CI: − 12.32 to − 3.84), both indicating greater improvement in the NW group. Quality of life, assessed by the WHOQOL-OLD, also favored the NW group (MD: 3.46; 95% CI: 0.84 to 6.09).

No clear between-group difference was observed for the TUG test (MD: − 0.40; 95% CI: − 1.15 to 0.36).

## Discussion

In the present study investigating the effectiveness of Nordic walking and traditional walking in older individuals, both methods were associated with improvements in lower extremity muscle strength and performance, aerobic capacity, balance, fall risk, and fear of moving. In addition, Nordic walking was associated with greater improvements in upper extremity muscle strength and performance. However, given the relatively small sample size and the presence of baseline imbalances, these findings should be interpreted with caution and considered preliminary.

This study also provides evidence regarding the feasibility of conducting such interventions in older adults. Recruitment was achievable, although relatively slow, and retention rates were acceptable and consistent with similar exercise-based interventions. The intervention was successfully delivered without adverse events, and all outcome measures were collected without missing data, supporting the feasibility of the study design.

Nordic walking is an exercise modality characterized by the active involvement of both the upper and lower extremities and may provide several potential benefits [7]. Previous studies have reported that 12 weeks of Nordic walking training resulted in significant improvements in lower extremity strength and performance, as assessed by the sit-to-stand test [16–21]. Similarly, in present study, regular walking training improved lower extremity muscle strength and performance; however, this improvement was significantly greater in the Nordic walking group. Furthermore, consistent with previous findings [16–18] and as expected, upper extremity muscle strength also increased in the Nordic walking group. This outcome may be attributed to the simultaneous activation of the upper and lower extremities during Nordic walking. Such simultaneous activation enhances body stability, allows for more effective load distribution on the lower extremities, increases overall muscle recruitment, improves neuromuscular coordination, and may have contributed to greater strength gains compared to traditional walking. Furthermore, the increase in upper extremity strength may be attributed to the isometric activation of the biceps during pole ground contact, particularly in the push-off phase of Nordic walking, and to the eccentric contraction occurring during muscle relaxation.

Research has shown that, compared to traditional walking, Nordic walking results in a higher heart rate and greater oxygen consumption [6]. In the present study, both walking modalities were found to be effective in improving aerobic capacity. This improvement can be attributed to the inherently aerobic nature of walking exercises, which stimulate cardiovascular and respiratory adaptations. Moreover, the improvements observed in favor of Nordic walking may be attributed to the activation of a greater muscle mass, increased energy expenditure, and higher cardiovascular load compared to traditional walking. Church et al. [22] also reported that, compared to normal walking, Nordic walking with poles promotes a faster walking speed. Although walking speed was not directly assessed in the present study, we consider that this factor may have contributed to the greater improvements observed in aerobic capacity. In our findings, both groups demonstrated improvements in TUG test performance; however, no clear between-group difference was identified. In contrast, some studies have reported that Nordic walking is effective in improving TUG performance in older adults [23, 24]. The literature suggests that 8 weeks of training is insufficient to improve TUG performance at the preferred speed. Indeed, even studies with 12-week interventions have shown only modest progress (approximately 12%), indicating that longer or more intensive training protocols may be required to achieve meaningful improvements in mobility [25]. Furthermore, the relatively normal baseline TUG values of our participants may also have contributed to this finding.

The use of poles in Nordic walking enhances balance and provides a more stable gait pattern [26]. Similarly, Andrejeva et al. reported that Nordic walking improves balance and reduces fall risk in older adults [27]. In line with these findings, our study demonstrated that both walking modalities had beneficial effects on fall efficacy and fear of moving, with greater improvements observed in the Nordic walking group. These results suggest that the additional support provided by the poles enabled participants to walk more safely and with greater confidence, thereby reducing their fear of movement. Thus, Nordic walking not only improved physical parameters but also contributed to overcoming psychological barriers, reducing fear-avoidance behaviors in older adults.

Walking interventions in older adults have been shown to positively influence different dimensions of quality of life, with multiple studies demonstrating improvements in various aspects of health and well-being [28]. In particular, Nordic walking has been associated with significant benefits in quality of life measures among older adults, including pain reduction, psychological well-being, and social functioning [29, 30]. Our study also revealed that both walking modalities had positive effects on quality of life in older individuals, largely attributable to the physical improvements they provided. Moreover, these improvements were more pronounced in the Nordic walking group. This may be explained by the additional physical benefits of Nordic walking, as well as its effects on reducing fear of moving and fall risk, thereby enhancing self-confidence and contributing to overall improvements in quality of life in older adults.

One strength of our study is that it evaluated Nordic walking in older adults not only on functional parameters but also on fall risk, fear of moving, and quality of life. Another strength is that, unlike most geriatric studies conducted indoors, our intervention was carried out combination of indoors and outdoors under physiotherapist supervision, which is particularly relevant in the post-COVID-19 period. Although adverse weather occasionally posed challenges, overall participant satisfaction was high. The relatively longer intervention duration compared to similar studies is also a strength. The limitations of this study include the absence of a participant satisfaction assessment and the inability to achieve the sample size initially estimated by the power analysis. In addition, the use of an active comparator (traditional walking) without a non-active control group may have limited the estimation of intervention-specific effect sizes. Furthermore, most outcomes assessed were intermediate measures, and higher-level functional outcomes such as activities of daily living and direct gait speed were not evaluated. Although recruitment, retention, and adherence were favorable, feasibility outcomes were not formally predefined. In addition, some aspects of the statistical analysis were refined after the initial study protocol, including the use of adjusted analyses and the increased emphasis on feasibility-related considerations. While these modifications were made to improve the robustness and interpretability of the findings, they were not pre-specified and should therefore be interpreted with caution. A lack of long-term follow-up limits conclusion regarding the sustainability of the observed effects. Future studies with larger, adequately powered samples, longer follow-up periods, and the inclusion of a non-active control group or a three-arm design are warranted to better determine the specific effects of Nordic walking, alongside functional and psychosocial outcomes.

## Conclusion

The present study indicates that both Nordic walking and traditional walking are associated with improvements in lower extremity muscle strength and performance, aerobic capacity, balance, fall efficacy, fear of moving, and quality of life in older adults. However, Nordic walking provided additional benefits, particularly by enhancing upper extremity strength, improving functional outcomes, and contributing to greater gains in functionality, self-confidence, and well-being. Taken together, our findings suggest that Nordic walking is a safe, feasible, and more effective alternative to traditional walking for promoting physical function, reducing fear-related barriers, and improving overall quality of life in the older adult population.

## Data Availability

De-identified data are available from the corresponding author upon reasonable request.
